# Enhanced visibility graph for EEG classification

**DOI:** 10.3389/fnins.2025.1541062

**Published:** 2025-05-27

**Authors:** Asma Belhadi, Pedro G. Lind, Youcef Djenouri, Anis Yazidi

**Affiliations:** ^1^Department Computer Science, Oslo Metropolitan University, Oslo, Norway; ^2^School of Economics, Innovation and Technology, Kristiania University of Applied Sciences, Oslo, Norway; ^3^Simula Research Laboratory, Numerical Analysis and Scientific Computing, Oslo, Norway; ^4^Department of Microsystems, University of South-Eastern Norway, Kongsberg, Norway; ^5^DARWIN, Norwegian Research Center NORCE, Oslo, Norway; ^6^Institute of Informatics, Oslo University, Oslo, Norway

**Keywords:** EEG classification, visibility graph, feature learning, deep learning, disease detection

## Abstract

Electroencephalography (EEG) holds immense potential for decoding complex brain patterns associated with cognitive states and neurological conditions. In this paper, we propose an end-to-end framework for EEG classification that integrates power spectral density (PSD) and visibility graph (VG) features together with deep learning (DL) techniques. Our framework offers a holistic approach for capturing both frequency-domain characteristics and temporal dynamics of EEG signals. We evaluate four DL architectures, namely multilayer perceptron (MLP), long short-term memory (LSTM) networks, InceptionTime and ChronoNet, applied to several datasets and in different experimental conditions. Results demonstrate the efficacy of our framework in accurately classifying EEG data, in particular when using VG features. We also shed new light on the relative strengths and limitations of different feature extraction methods and DL architectures in the context of EEG classification. Our work contributes to advancing EEG analysis and facilitating the development of more accurate and reliable EEG-based systems for neuroscience and beyond. The full code of this research work is available on https://github.com/asmab89/VisibilityGraphs.git.

## 1 Introduction

In recent years, the field of electroencephalography (EEG) has witnessed significant advancements, fueled by the growing demand for non-invasive methods to understand brain activity and cognitive processes (Bao et al., 2023; Fiscon et al., 2018). EEG signals, which represent the electrical activity of the brain recorded from the scalp, offer a rich source of information for various applications ranging from medical diagnosis (Tawhid et al., 2023) to brain-computer interface systems (Khan et al., 2023) and even decoding brain-computer interfaces (BCIs) (Dreyer et al., 2023). Among the plethora of applications, EEG-based classification tasks have garnered substantial attention due to their potential to decode complex brain patterns associated with different cognitive states, mental disorders and neurological conditions (Chen et al., 2023; Ali et al., 2024; Chen et al., 2024). Classifying EEG signals poses a significant challenge due to their inherent complexity, non-stationarity and high dimensionality (Anuragi et al., 2024). It also involves distinguishing between different cognitive states or identifying anomalies indicative of neurological conditions (Altaheri et al., 2023). Traditional approaches often rely on handcrafted features extracted from the EEG signals to characterize distinct patterns associated with different mental states or neurological conditions (Amin et al., 2015; Wang et al., 2014; Hosseini et al., 2020). However, these methods are often limited by their reliance on predefined features and may not fully capture the underlying dynamics of the EEG signals. In response to these challenges, advanced ML represented by the deep learning has emerged as a powerful tool for automatic feature extraction and classification in EEG analysis (Liu et al., 2024; Luo et al., 2024). Convolutional Neural Networks (CNNs), in particular, have demonstrated promising results in various signal processing tasks (Jalagam and Mittal, 2024). However, EEG signals possess unique characteristics, such as non-stationarity, non-linearity and temporal dependencies, which may not be effectively captured by standard CNN architectures.

**Motivation:** While existing EEG classification methods–from spectral analysis to deep learning–have demonstrated competence in specific applications (Wang et al., 2023; Chen et al., 2021; Gemein et al., 2020), their broader utility faces two main challenges. First, conventional approaches often struggle to model the non-linear temporal dynamics inherent in neural signals, treating EEG as piecewise stationary or relying on short-term spectral features that may miss critical phase-space relationships. Second, real-world EEG variability (e.g., noise, artifacts) continues to degrade performance, as most algorithms are optimized for controlled experimental conditions. Our visibility graph framework addresses these limitations simultaneously by (1) encoding temporal dependencies through network topology, and (2) providing graph-derived biomarkers aligned with known neural phenomena (e.g., small-world properties during cognitive load). This work thus fills a critical gap between signal-processing theory and clinical applicability, where existing solutions remain insufficient. This research addresses this need by exploring and comparing two innovative feature extraction methods: PSD (Alsolamy and Fattouh, 2016) and VG (Lacasa et al., 2008). The PSD method is instrumental in providing detailed insights into the frequency-domain characteristics of EEG signals. By focusing on spectral information, PSD helps in distinguishing different brain states, which is pivotal for accurate EEG classification. On the other hand, the Visibility Graph approach introduces a transformative perspective by converting EEG time series into complex networks. By complex networks, we refer to networks formed by the relationships between frequency-domain features derived from PSD of the EEG signal. This method leverages graph-theoretical measures to uncover intricate patterns and relationships within the data that traditional methods might overlook. The novelty of VG lies in its ability to encapsulate the temporal structure and connectivity of EEG signals, offering a unique dimension to feature extraction that complements the frequency-based insights of PSD. To further enhance the classification accuracy, this paper investigates the performance of four cutting-edge deep learning architectures: MLP (Basha et al., 2020), LSTM (Yu et al., 2019), InceptionTime (Ismail Fawaz et al., 2020), and ChronoNet (Roy et al., 2019). Each architecture brings distinct advantages: MLP provides a baseline for understanding the non-temporal features, LSTM excels in modeling temporal dependencies, InceptionTime captures hierarchical patterns efficiently, and ChronoNet integrates temporal and frequency-domain features seamlessly. By comparing these architectures with the proposed feature extraction methods, this research highlights the strengths and limitations of each combination. The analysis contributes to developing more robust EEG classification systems, with implications for brain-computer interfaces, neurofeedback, and clinical diagnostics. The findings pave the way for novel methodologies to better interpret complex EEG data, advancing both theoretical and applied neuroscience (Dong et al., 2023). While deep learning has shown promise in EEG analysis, its generalizability remains constrained by variability in acquisition protocols, subject demographics, and recording conditions. Our framework bridges this gap through a principled fusion of deep learning with physiologically-grounded graph features. The integration of visibility graph-derived topological metrics—which are inherently robust to amplitude variations—with learned representations creates a more generalizable architecture. Cross-dataset validation demonstrates our approach's superior robustness, maintaining stable performance where conventional DL models exhibit significant degradation. Although perfect generalization remains elusive, our hybrid paradigm represents a meaningful advance toward clinically-reliable EEG classification.

**Contributions:** The main contributions of this research work might be listed as follows:

1. We propose an end-to-end framework for EEG classification that seamlessly integrates feature learning and classification stages. By intelligently incorporating PSD and VG features, our framework offers a holistic approach to capturing both frequency-domain characteristics and temporal dynamics of EEG signals.

2. We systematically compare the performance of PSD and VG features for EEG classification task. By evaluating these methods across multiple datasets and experimental conditions, we provide insights into their relative strengths and limitations in capturing discriminative information from EEG signals.

3. We conduct a comparative study of four deep learning architectures tailored for EEG classification: MLP, LSTM, InceptionTime and ChronoNet. Through extensive experiments, we analyze their performance in terms of accuracy to classify EEG data.

**Paper Outline:** The paper is organized as follows. Section 2 formulates the EEG classification problem. Section 3 reviews the existing solutions for EEG classification. Section 4 describes our methodology of incorporating PSD and VG features in four deep learning architectures. Section 5 presents the performance evaluation of our designed methodology, followed by drawing future directions in Section 6. Section 7 concludes the paper.

## 2 The problem of EEG classification: the general formulation

**Figure 1:**
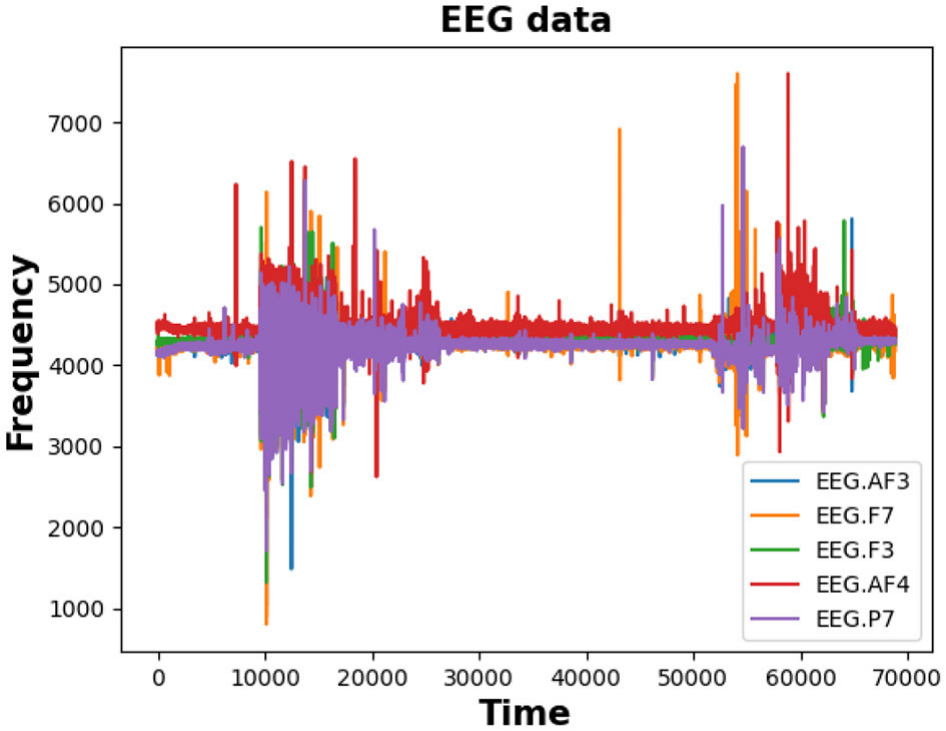
EEG data illustration: an example of EEG data containing five channels: AF3, F7, F3, AF4, and P7.

Consider a multiple-channel EEG signal $X=\left\{X_{1},X_{2},...,X_{n}\right\}$. Each EEG sample *X*_*i*_ encompasses *n* channels, with *n* denoting the number of electrodes or sensors utilized for capturing brain signals. The primary aim of EEG data classification is to assign a label *y*_*i*_ from a predetermined set of classes to each EEG sample *X*_*i*_, indicating the corresponding brain state or activity. Formally, let *Y* = {*y*_1_, *y*_2_, ..., *y*_*n*_} denote the labels associated with the EEG samples, with each label *y*_*i*_ drawn from a predefined set of classes *C* = {*c*_1_, *c*_2_, ..., *c*_*k*_}. The task of EEG data classification involves learning a mapping function *f*:*X*→*Y*, which accurately assigns the appropriate class label *y*_*i*_ to each EEG sample *X*_*i*_ based on its brain activity patterns. The classification process entails training a model using a labeled dataset *D* = {(*X*_1_, *y*_1_), (*X*_2_, *y*_2_), ..., (*X*_*n*_, *y*_*n*_)}, where each EEG sample *X*_*i*_ is paired with its corresponding label *y*_*i*_. The model is trained to minimize a chosen loss function, such as cross-entropy, while optimizing the model parameters to enhance classification accuracy. Once trained, the model can predict the class labels of new, unseen EEG samples, enabling real-time or offline classification of brain activity. Evaluation of the EEG data classification model typically involves metrics such as accuracy, precision, recall, and F1 score computed on a separate validation or test dataset. The overarching goal of EEG data classification is to enable precise and automated analysis of brain activity, fostering applications in areas like brain-computer interfaces, sleep monitoring, neurology diagnosis, and cognitive research. Figure 1 plots EEG data from five channels (AF3, F7, F3, AF4, and P7) to explore the correlation between course comprehension and EEG signal patterns.

EEG classification is typically NP-hard or NP-complete, reflecting its computational complexity. This challenge arises from the high dimensionality of EEG signals, which involve multiple channels sampled over time, complicating feature extraction and selection. The temporal dynamics of these signals, which include long-range dependencies, further increase the complexity (Karamzadeh et al., 2013). Additionally, the non-linear relationships between EEG features and cognitive processes demand advanced machine learning techniques (Stam et al., 1996). Consequently, EEG classification often requires sophisticated optimization approaches to find feasible solutions efficiently.

## 3 Related work

Lawhern et al. (2018) presented EEGNet, a compact convolutional neural network architecture specifically designed for EEG-based BCI. The model incorporates depthwise and separable convolutions to create an EEG-optimized framework that inherently captures established BCI feature extraction principles. Rivet et al. (2009) presented, xDAWN algorithm, an unsupervised algorithm for enhancing P300 evoked potentials through optimized spatial filter estimation. The method projects raw EEG signals into a derived signal subspace to improve feature separability. Tibermacine et al. (2024) presented a framework for EEG signal classification that combines Riemannian geometry with a custom contrastive learning approach. The method begins by segmenting EEG signals and transforming them into regularized covariance matrices, ensuring positive definiteness for representation as symmetric positive definite (SPD) matrices. These SPD matrices are then mapped onto a Riemannian manifold, where discriminative features are extracted through geometric operations in the tangent space. The framework introduced a TangentSpaceNet, a specialized neural network architecture that projects these features into a lower-dimensional embedding space. Several approaches have explored visibility graphs for EEG classification. Sudhamayee et al. (2023) proposed a simplicial method where cliques in visibility graphs are treated as simplices to capture both broad and localized features in time series data. Nasrolahzadeh et al. (2023) applied visibility graphs to analyze Alzheimer's Disease speech dynamics using complexity and fractality metrics. Kutluana and Türker (2024) leveraged node weights or adjacency matrix diagonals from visibility graphs as features for ResNet and Inception models, reducing graph dimensionality. Jain and Ganesan (2021) used visibility graphs and temporal features to classify sleep stages, integrating graph-based metrics with autoregressive model coefficients and fractal dimensions. Cai et al. (2020) introduced multiplex visibility graph motifs and a CNN for sleep stage classification, while Wadhera and Mahmud (2022) developed a two-layered Visible-Graph Convolutional Network (VGCN) mapping EEG samples onto nodes. Samanta et al. (2019) utilized multiplex weighted visibility graphs for EEG-based brain connectivity networks, incorporating autoencoder-based feature extraction. Kong et al. (2021) extracted features from forward and backward weighted horizontal visibility graphs for emotion recognition, combining them into a unified feature matrix.

Current EEG classification solutions often fail to efficiently extract relevant features, limiting classification accuracy. To overcome this, we propose a hybrid method that combines PSD and VG features with advanced deep learning architectures. PSD features capture frequency-domain information, while VG features convert EEG time series into complex networks, revealing hidden patterns through graph-theoretical analysis. This integration results in a richer feature set for EEG classification. We also leverage deep learning models like MLP, LSTM, InceptionTime, and ChronoNet to effectively capture temporal dependencies and enhance classification performance.

## 4 A general framework for EEG classification

**Figure 2:**
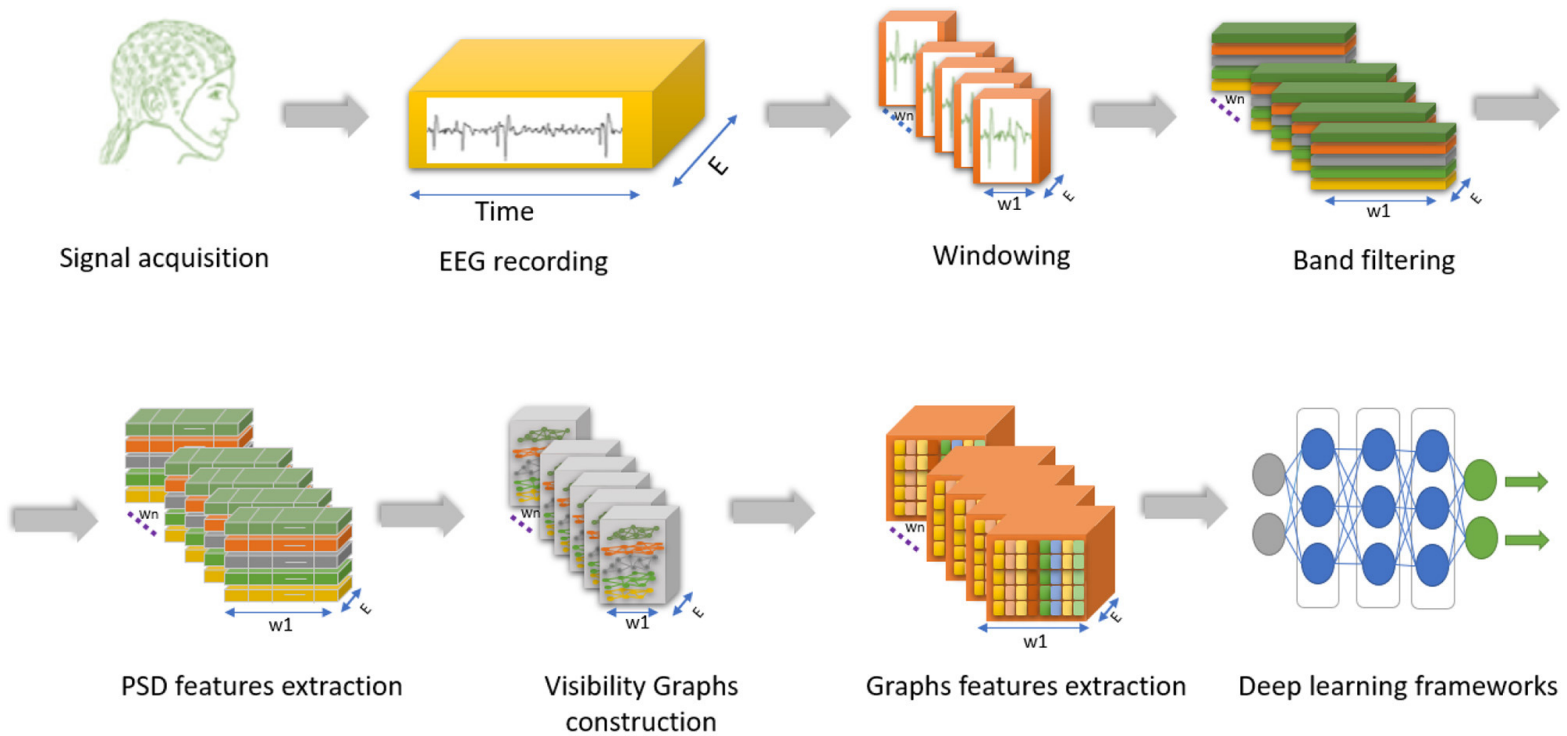
General framework of PSD-based solution for EEG classification. The described steps include segmenting the EEG time series, isolating frequency bands, extracting PSD features, constructing visibility graphs to depict the intrinsic neural dynamics, deriving graph features to capture network topology and inputting them into deep learning models.

The proposed model, illustrated in Figure 2, comprises multiple components. The process begins with segmenting the EEG time series into windows. Each window undergoes bandpass filtering to isolate specific frequency bands of interest, allowing for a focused examination of neural oscillations. Next, we extract PSD features from each frequency band, which capture the distribution of signal power across different frequencies. The PSD features form the basis for constructing visibility graphs, providing a graphical representation of the underlying neural dynamics. Each graph reflects the structural relationships among EEG data points within the corresponding frequency band. From these visibility graphs, we derive a comprehensive set of graph features, encapsulating essential characteristics of network topology. These graph features are then fed into a deep learning architecture. We employ various deep learning models, including MLP, LSTM, InceptionTime, and ChronoNet, to analyze the data. Each of these steps is detailed in the following sections.

### 4.1 Preprocessing and PSD features creation

We conducted minimal preprocessing on each raw recording, which involved only the referencing step. It is a fundamental preprocessing procedure aimed at standardizing the recorded signals and removing biases or artifacts that could obscure the underlying neural activity of interest. This process involves adjusting each electrode's signal relative to a reference point to isolate the brain's electrical activity from common-mode signals shared across electrodes. Afterwards, we partitioned the preprocessed EEG recordings into continuous, non-overlapping windows. Each window contains EEG data collected from all channels. The following five frequency bands are then calculated from each created EEG segment, namely δ-band (0.5–4 Hz), θ-band (4–8 Hz), α-band (8–12 Hz), β-band (12–30 Hz) and γ-band (30–100 Hz). Each frequency band in EEG signals corresponds to specific ranges that reflect different aspects of brain activity. For example, delta waves indicate deep sleep and unconscious processing, theta waves are present during relaxation and light sleep, alpha waves are prominent during wakeful relaxation and the resting state, and beta waves are associated with active thinking and cognitive engagement. Gamma waves, characterized by higher frequencies, are involved in cognitive processes like attention, memory formation, and perception. Segmenting EEG data into these frequency bands allows us to analyze unique neural dynamics that underlie various states of consciousness and cognitive tasks. The final step in this process is to generate the PSD features. We apply the Welch method (Welch, 1967) to extract PSD features from each segment of EEG data. This method divides the EEG data into overlapping windows and computes the Fourier transform for each window. By averaging these transforms, the Welch method offers a more reliable PSD estimate compared to conventional approaches, especially in scenarios involving sparse data or non-stationary signals. The resulting PSD illustrates how power is distributed across various frequency ranges within each EEG segment.

### 4.2 Graph construction and embedding

The concept of visibility graphs was initially introduced by Lacasa et al. (2008) as a technique for transforming time series data into network structures. In their approach, individual time points within the sequential data act as nodes in the graph representation, while the visibility criteria between these nodes dictate the establishment of edges in the graph. Visibility graphs are particularly useful for analyzing patterns in time series data, identifying trends and detecting significant events. They provide a graphical representation that can aid in the visualization and understanding of complex temporal relationships within the data. In our approach, we adopt a methodology where visibility graphs are created on the top of the PSD features. Within this framework, every individual power point found within the designated frequency spectrum acts as a node in the resultant graph representation. Notably, the establishment of edges between sequential PSD points relies on the absence of any intervening bars, signifying unimpeded visibility between them (if no other bar is blocking visibility). In a more formal sense, a visibility graph G constructed based on PSD features is a tuple (V,E) where V is the set of nodes representing the sequence of PSD data points and E is the set of edges representing visibility relationships between nodes. The formal definition of visibility relationships within this context is articulated as follows: Given a sequence of sequential PSD (2-dimensional) data points *P*_*i*_, two PSD data points *P*_*i*_ = (*s*_*i*_, *y*_*i*_) and *P*_*j*_ = (*s*_*j*_, *y*_*j*_) are connected if any other PSD data point *P*_*k*_ = (*s*_*k*_, *y*_*k*_) placed between them satisfies the so-called visibility criterion:


(1) $$\begin{array}{l} y_{k}<y_{j}+\left(y_{i}-y_{j}\right)\frac{s_{j}-s_{k}}{s_{j}-s_{i}}. \end{array}$$

**Figure 3:**
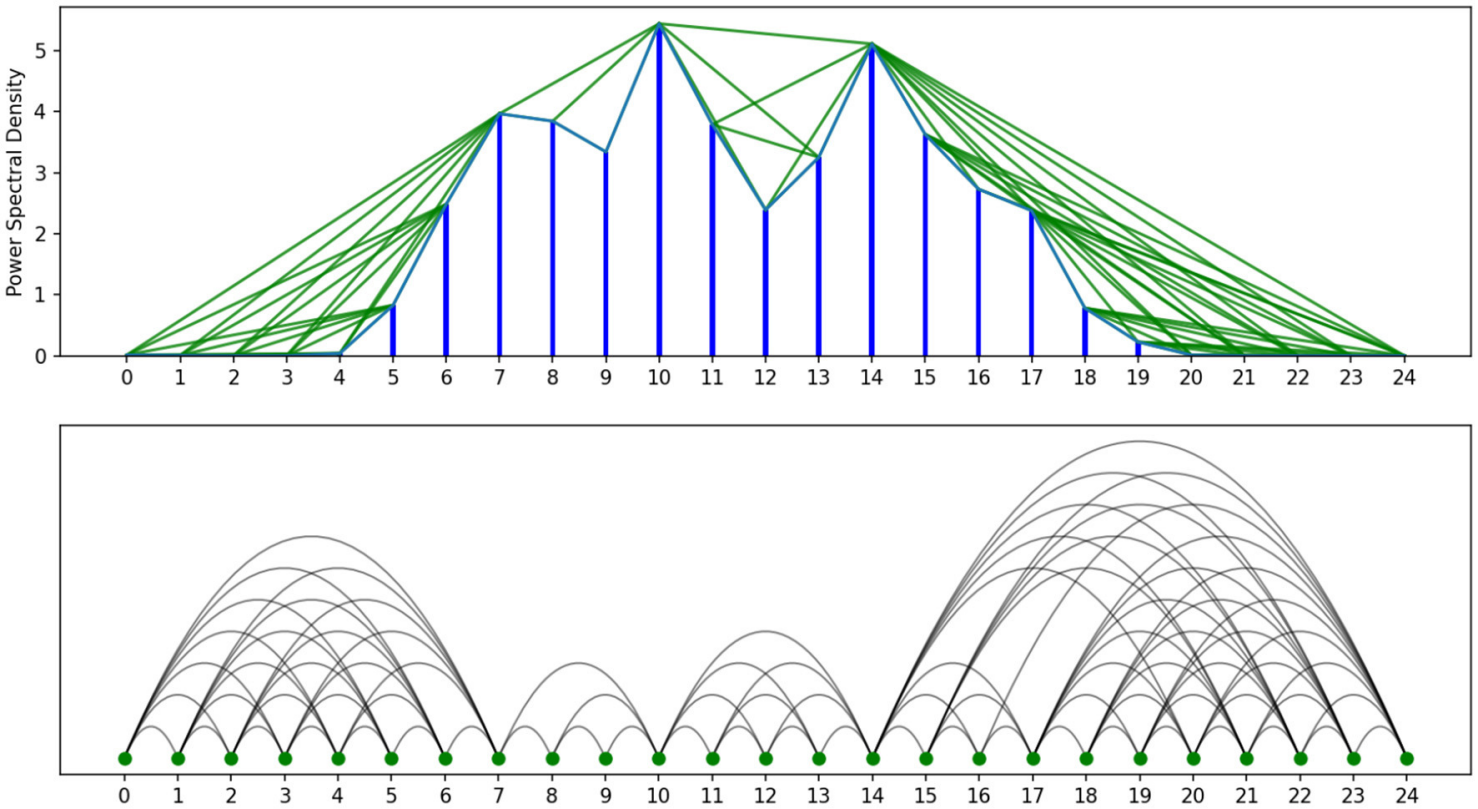
Illustration of the PSD feature transformation into a visibility graph using a specific channel and frequency band segment from the Schizophrenia dataset (Section 5). A green line between two data points indicates mutual visibility, connecting the corresponding vertices with an edge.

Figure 3 illustrates the transformation of PSD features into a visibility graph. By running the same procedure for all PSD data points, a graph representation is generated for the whole sequential data. We then used distinct strategies to compute the embedding features of the created graph. By quantifying key characteristics of the network, these metrics serve as valuable features for subsequent classification tasks, enabling the identification of distinct patterns or classes within the data.

### 4.3 Graph embedding strategies

In this section, we illustrate the graph embedding strategies used by incorporating several metrics to analyze and represent graph structures. These strategies include the average degree, which is computed as:


(2) $$\begin{array}{l} \bar{d}=\frac{1}{n}\overset{n}{\underset{i=1}{\sum }}d_{i} \end{array}$$

where *d*_*i*_ is the degree of vertex *i* and *n* is the total number of vertices. This metric provides insight into the typical connectivity of nodes.

The maximum degree, Δ, measures the highest number of connections any vertex has, while graph density, D(G), quantifies how close the graph is to being fully connected. They are defined as:


(3) $$\begin{array}{l} \text{Δ}=\underset{v\in V}{\max}\deg\left(v\right) \end{array}$$

and,


(4) $$\begin{array}{l} D\left(G\right)=\frac{2|E|}{|V|\left(|V|-1\right)} \end{array}$$

We calculate the radius, *r*, which measures the smallest maximum distance from any vertex to all others, and the diameter, *D*, representing the longest shortest path between any two vertices. They are defined as:


(5) $$\begin{array}{l} r=\underset{v\in V}{\min}\underset{u\in V}{\max}d\left(u,v\right) \end{array}$$

and,


(6) $$\begin{array}{l} D=\underset{v\in V}{\max}\underset{u\in V}{\max}d\left(u,v\right) \end{array}$$

Equation 5 calculates the radius (r), which measures the smallest maximum distance from any vertex v to all other vertices u in the graph. In contrast, Equation 6 calculates the diameter (D), which represents the longest shortest path between any two vertices in the graph. Both equations involve the distance function d(u,v), but the radius focuses on minimizing the maximum distance from a specific vertex, whereas the diameter looks for the maximum distance across all pairs of vertices in the graph.

We measure the uncertainty in the graph's structure by computing the entropy,


(7) $$\begin{array}{l} H\left(G\right)=-\overset{n}{\underset{i=1}{\sum }}p_{i}\log\left(p_{i}\right)-\overset{n}{\underset{i=1}{\sum }}q_{i}\log\left(q_{i}\right) \end{array}$$

In Equation 7, we compute the entropy *H*(*G*) to measure the uncertainty in the graph's structure, which is based on the probability distributions associated with the graph's properties. Specifically, *p*_*i*_ and *q*_*i*_ represent probability distributions over certain features or characteristics of the graph, such as node degrees, edge weights, or any other relevant metric in the context of graph structure. For example, *p*_*i*_ could represent the probability distribution over node degrees, and *q*_*i*_ could represent the distribution over another graph property (e.g., clustering coefficients).

We also introduce a global efficiency that measures the efficiency of information exchange across the network, and defined as:


(8) $$\begin{array}{l} E_{global}\left(G\right)=\frac{1}{n\left(n-1\right)}\underset{u\neq v}{\sum }\frac{1}{d\left(u,v\right)} \end{array}$$

Finally, the maximum clique size that indicates the size of the largest fully connected subset of vertices. It is defined as,


(9) $$\begin{array}{l} \omega \left(G\right)=\max\left\{|C|:C\text{is a clique in}G\right\} \end{array}$$

**Theorem 1:** Let G=(V,E) be a connected graph with radius *r* and diameter *D*. The following relationships hold: 1. *D* ≤ 2*r* 2. *r* ≤ *D*

*Proof*. **Relation 1:** The diameter *D* is defined as:


(10) $$\begin{array}{l} D=\underset{u,v\in V}{\max}d\left(u,v\right), \end{array}$$

where *d*(*u, v*) denotes the shortest path distance between vertices *u* and *v*. The radius *r* is defined as:


(11) $$\begin{array}{l} r=\underset{u\in V}{\min}\underset{v\in V}{\max}d\left(u,v\right). \end{array}$$

Let u^*^ be a vertex where the radius *r* is achieved. For any vertex *v* in G, we have:


(12) $$\begin{array}{l} d\left(u^{*},v\right)\leq r. \end{array}$$

Consider any two vertices *x* and *y*. The shortest path *d*(*x, y*) can be bounded by:


(13) $$\begin{array}{l} d\left(x,y\right)\leq d\left(x,u^{*}\right)+d\left(u^{*},y\right). \end{array}$$

Since *d*(x, u^*^) ≤ *r* and *d*(u^*^, *y*) ≤ *r*, we get:


(14) $$\begin{array}{l} d\left(x,y\right)\leq d\left(x,u^{*}\right)+d\left(u^{*},y\right)\leq r+r=2r. \end{array}$$

Therefore:


(15) $$\begin{array}{l} D\leq 2r. \end{array}$$

**Relation 2:** Since the radius *r* is defined as:


(16) $$\begin{array}{l} r=\underset{u\in V}{\min}\underset{v\in V}{\max}d\left(u,v\right), \end{array}$$

it follows that for any vertex *u*, the maximum distance from *u* to any other vertex is at most *r*. Hence:


(17) $$\begin{array}{l} \underset{v\in V}{\max}d\left(u,v\right)\leq r. \end{array}$$

Thus, the diameter *D*, which is the maximum distance between any pair of vertices, must be at least as large as this maximum distance:


(18) $$\begin{array}{l} D\geq r. \end{array}$$

**Theorem 2**: Let G=(V,E) be a graph with diameter *D* and maximum clique size ω. The following relationship holds:


(19) $$\begin{array}{l} \omega \leq \frac{D+1}{2}. \end{array}$$

*Proof*. Consider a maximum clique *C* in G with size ω. Since every vertex in *C* is connected to every other vertex, the distance between any two vertices within *C* is at most 1.

For any vertex *v* ∈ *V* not in *C*, the distance between *v* and any vertex in *C* is at most *D*. Therefore, if *v* is connected to all vertices in *C*, the size of *C* is limited by the maximum distance *D* as:


(20) $$\begin{array}{l} \omega \leq \frac{D+1}{2}. \end{array}$$

This is because the clique *C* must be large enough to cover the diameter of the graph with minimal overlap in connections, which is bounded by $\frac{D+1}{2}$.

### 4.4 Deep learning framework structure and implementation

After extracting the graph features, we developed four main classes of deep learning models for EEG classification. The first one is MLP (Basha et al., 2020) which provides a simple and straightforward approach for capturing global patterns in the data, while the second one is LSTM (Yu et al., 2019) that excels in capturing long-term dependencies crucial for understanding temporal dynamics in EEG signals. The third one is InceptionTime (Ismail Fawaz et al., 2020) that leverages convolutional neural network (CNN) architectures to efficiently extract multi-scale features, enabling the identification of both local and global patterns in the data. The last one is ChronoNet (Roy et al., 2019) which combines convolutional and recurrent layers to capture both spatial and temporal dependencies simultaneously. The detailed description of the four DL architectures used in this research work is given as follows:

1. **Multilayer perceptron (MLP)**: It is a fully-connected neural network consisting of six densely connected layers, each containing 64 neurons. Throughout these layers, we utilized the Rectified Linear Unit (ReLU) Activation Function, commonly employed in hidden layers of neural networks. The output layer, tailored for binary classification tasks, comprises a single neuron with a sigmoid activation function.

2. **Long short-term memory (LSTM)** models: It is composed of three layers. The first and second layers consist of 100 and 50 units, respectively, with both configured to return sequences. Batch normalization is applied after the first layer for stabilization during training. A dropout layer with a dropout rate of 20% is incorporated after the second layer for regularization. The third layer, comprising 25 units, is the final layer and does not return sequences. The output layer is a single neuron with a sigmoid activation function.

3. **InceptionTime**: The InceptionTime architecture utilizes multiple convolutional filters with different kernel sizes (1, 3 and 5) in parallel to capture features at various temporal resolutions efficiently. Each inception module consists of several convolutional layers with different kernel sizes, followed by an average pooling layer and a 1x1 convolutional layer. These layers are concatenated along the channel axis to form a rich representation of the input data. The architecture stacks multiple inception modules, progressively increasing the number of filters to capture more complex temporal patterns. Finally, a flattening layer is applied to transform the multi-dimensional feature maps into a one-dimensional vector, followed by a dense layer with sigmoid activation to output class probabilities for the EEG classification task.

4. **ChronoNet**: This architecture begins with a block function that consists of three parallel convolutional layers with different kernel sizes and strides, allowing the model to capture diverse temporal features effectively. These convolutional layers utilize the ReLU Activation Function for introducing non-linearity and are concatenated along the channel axis to create a richer representation of the input data. The output of this block is then fed into multiple Gated Recurrent Unit (GRU) layers, a type of recurrent neural network known for capturing temporal dependencies in sequential data. These GRU layers are stacked to enable the model to learn hierarchical representations of the input sequence. Finally, a dense layer with a sigmoid activation function is applied to produce the final prediction. This architecture is designed to handle irregularly sampled time series data.

## 5 Performance evaluation

### 5.1 Datasets and metrics

In this section, we provide an overview of the datasets, metrics and baseline models utilized for evaluation.

1. **Schizophrenia dataset**: The research involved a group of 14 individuals diagnosed with paranoid schizophrenia (with an equal distribution of 7 males and 7 females). These patients were receiving hospital care at the Institute of Psychiatry and Neurology in Warsaw, Poland. Additionally, a control group consisting of 14 healthy individuals, (with an equivalent distribution of 7 males and 7 females) was also included in the study. The study protocol received approval from the Ethics Committee of the Institute of Psychiatry and Neurology in Warsaw, as it is reported in the original study, by the team which collected the data. For details see Olejarczyk and Jernajczyk (2017). A 15-minute EEG data recording was conducted on all subjects during a resting state with eyes closed. The data were acquired at a sampling frequency of 250 Hz using the standard 10 − 20 EEG montage, comprising 19 EEG channels: *Fp*1, *Fp*2, *F*7, *F*3, *Fz*, *F*4, *F*8, *T*3, *C*3, *Cz*, *C*4, *T*4, *T*5, *P*3, *Pz*, *P*4, *T*6, *O*1, *O*2. The reference electrode was placed at *FCz*.
2. **Guinea-Bissau epilepsy dataset**: A total of 97 participants from Guinea-Bissau were included in the study. Approval for the study in Guinea-Bissau was obtained from organizational boards as well as local and national government bodies, as stated in the original study conducted by the data collection team (refer to van Hees et al., 2018 for details). A 5-minute resting-state EEG data was recorded using a portable, low-cost consumer-grade EEG recording headset equipped with 14 channels: *AF*3, *AF*4, *F*3, *F*4, *F*7, *F*8, *FC*5, *FC*6, *O*1, *O*2, *P*7, *P*8, *T*7 and *T*8 following the International 10 − 20 system. The EEG configuration was configured to sample at 128 Hz. Participants were instructed to sit on a chair for 5 minutes while wearing the wireless headset. For the study, 2 minutes of resting-state EEG data were recorded with closed eyes during this 5-minute period.
3. **Intellectual and developmental disabled dataset:** The dataset consists of 14 subjects, with 7 diagnosed as having intellectual and developmental disabilities (IDD) (all male, aged between 26 and 31 years) and 7 typically developing control (TDC) subjects (all male, aged between 18 to 56 years. The Intelligence Quotient (IQ) of IDD subjects ranges from 52 to 68 and the Social Quotient (SQ) ranges from 57 to 62. Ethical approval for the experiment was obtained from the Institute's Ethics Committee and the study was conducted in accordance with the ethical standards outlined in the Declaration of Helsinki, as outlined in the original study by the data collection team (see Sareen et al., 2020 for further details). EEG signals are recorded with a sampling frequency of 128 Hz from 14 electrodes placed on the scalp according to the 10 − 20 international system. The device's channel configuration includes: *AF*3, *F*7, *F*3, *FC*5, *T*7, *P*7, *O*1, *O*2, *P*8, *T*8, *FC*6, *F*4, *F*8 and *AF*4. Data for each subject includes a 2-minute recording under rest conditions followed by a 2-minute exposure to music stimuli.

Table 1 provides a summary of the datasets utilized in the experiments. We evaluated our model with standard EEG classification metrics: accuracy, precision, recall, and F1-score, which are described as follows:

- **Accuracy**: It measures the proportion of correct predictions in relation to the total number of samples. In general, a higher ratio indicates better performance where a value of 100% means that all predictions made by the model were correct.
- **Precision**: It is defined as the percentage of samples predicted as positive that were truly positive. A higher precision value indicates more accurate positive class predictions. The precision criterion is outlined as follows.

(21) $$\begin{array}{l} \text{precision}=\frac{TP}{TP+FP}, \end{array}$$

where *TP* is the number of true positives and *FP* is the number of false positives.

- **Recall**: It is the ratio of all positive samples correctly predicted to all actual positive samples. A higher recall value indicates the ability to capture more positive class samples from the entire set of positive samples. The recall is articulated as follows.

(22) $$\begin{array}{l} \text{recall}=\frac{TP}{TP+FN}, \end{array}$$

where *FN* is the number of false negatives.

- **F1 score**: It is the harmonic mean of precision and recall. A higher F1 score indicates a better balance between precision and recall, while a lower F1 score suggests an imbalance between the two metrics. It is calculated using the following formula:

(23) $$\begin{array}{l} F1=2*\frac{\text{Precision}\times \text{Recall}}{\text{Precision}+\text{Recall}}. \end{array}$$

**Table 1:** Datasets Description.

**Dataset name**	**# Individuals**	**#Control individuals**	**# Individuals with disease**	**Balanced/unbalanced**
Schizophrenia	14	7	7	Balanced
Epilepsy	97	46	51	Unbalanced
IDD	14	7	7	Balanced

To mitigate overfitting, we used five-fold cross-validation, where the dataset was split into five subsets. In each fold, four subsets were used for training and one for testing, with each subset being tested once. This process was repeated ten times, and the final metrics were averaged across all runs. This approach ensures a robust, unbiased evaluation and improves the model's generalizability.

### 5.2 Results

**Figure 4:**
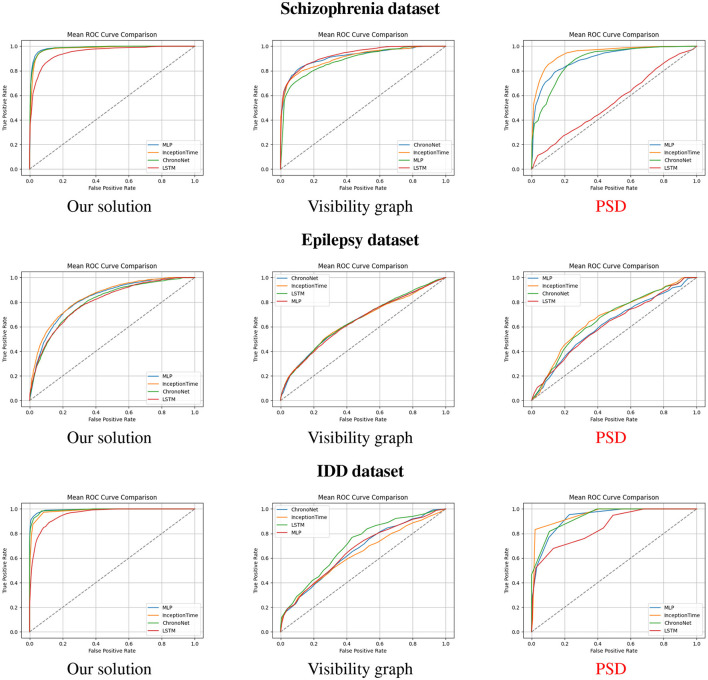
The mean ROC curves of our solution compared to visibility graph and PSD based solutions using different datasets.

We conducted a comprehensive series of experiments to rigorously evaluate the effectiveness of our proposed framework, which integrates PSD features with VG representations for EEG classification. To ensure robustness and statistical validity, we implemented a repeated evaluation strategy involving 10 iterations of 5-fold cross-validation for each model and dataset. Performance was assessed using standard classification metrics–accuracy, precision, recall, and F1-score–as presented in Tables 2, 3. Additionally, Figure 4 illustrates the averaged ROC curves across all iterations for four representative deep learning models: MLP, InceptionTime, ChronoNet, and LSTM, under different feature configurations (i.e., with PSD, with VG, and with both). Our experimental pipeline was applied across three neurologically diverse EEG datasets: schizophrenia, epilepsy, and individuals with IDD. Window sizes were empirically optimized for each dataset to best capture relevant temporal dynamics–30 s for schizophrenia and epilepsy, and 5 s for the IDD dataset, reflecting the different cognitive and neurophysiological profiles of the populations involved. The results were consistent and compelling across all three datasets. On the schizophrenia dataset, both MLP and InceptionTime achieved an accuracy of 0.95. For epilepsy, MLP achieved a peak accuracy of 0.76, highlighting the inherent difficulty of the task due to more subtle EEG patterns. The IDD dataset demonstrated the most promising results: MLP, InceptionTime, and ChronoNet all achieved an outstanding accuracy of 0.98. Crucially, the combined approach–integrating both PSD features and visibility graph representations–consistently outperformed models trained on either PSD or VG features alone. Notably, the AUC scores for the combined model exceeded 0.99 across all datasets, compared to significantly lower AUC values (as low as 0.70) when PSD features were excluded. In addition to AUC improvements, we observed substantial gains in F1-scores, with increases exceeding 10% in many configurations. These results underscore the synergistic value of combining frequency-domain information from PSD with the topological insights offered by VG analysis. Together, these features enrich the model's understanding of both spectral power distribution and temporal structural complexity, leading to superior classification performance. Despite these gains, we acknowledge the increased computational complexity introduced by this dual-feature strategy, which may pose challenges for real-time deployment. Moreover, performance gains were somewhat architecture- and dataset-dependent, suggesting that future research should explore more efficient model integration techniques and automated adaptation mechanisms. Ultimately, our findings demonstrate that the hybridization of PSD and VG features represents a powerful direction for EEG-based diagnostic modeling, with strong potential for generalization to broader neurocognitive classification tasks.

**Table 2:** Quality performance of the designed solution on the three datasets.

**Dataset**	**Algorithm**	**Accuracy**	**Precision**	**Recall**	**F1 score**
Schizophrenia	MLP	0.95	0.95	0.96	0.96
	LSTM	0.88	0.88	0.91	0.89
	ChronoNet	0.94	0.94	0.95	0.95
	InceptionTime	0.95	0.95	0.95	0.95
Epilepsy	MLP	0.75	0.74	0.73	0.73
	LSTM	0.72	0.70	0.70	0.70
	ChronoNet	0.73	0.71	0.68	0.70
	InceptionTime	0.76	0.76	0.70	0.73
IDD	MLP	0.98	0.98	0.98	0.98
	LSTM	0.90	0.90	0.90	0.90
	ChronoNet	0.98	0.98	0.98	0.98
	InceptionTime	0.98	0.98	0.98	0.98

**Table 3:** F1 score performance of the designed solution on the three datasets compared to visibility graph and PSD based solution.

**Dataset**	**Algorithm**	**Our solution**	**With only visibility graph**	**With only PSD**
schizophrenia	MLP	0.96	0.80	0.68
	LSTM	0.89	0.83	0.69
	ChronoNet	0.95	0.85	0.83
	InceptionTime	0.95	0.82	0.88
epilepsy	MLP	0.73	0.58	0.68
	LSTM	0.70	0.58	0.68
	ChronoNet	0.70	0.58	0.69
	InceptionTime	0.73	0.58	0.66
IDD	MLP	0.98	0.60	0.75
	LSTM	0.90	0.57	0.76
	ChronoNet	0.98	0.59	0.80
	InceptionTime	0.98	0.57	0.93

## 6 Future directions

1. **Noisy and artifactual processing**: A major challenge in EEG classification is handling noise and artifacts from environmental interference, muscle activity, and electrode artifacts. These disturbances degrade EEG signal quality, hindering classification performance. EEG signals can be contaminated by electrical noise from equipment or power lines, obscuring brain activity with high-frequency noise or baseline shifts. Effective denoising and filtering techniques are critical to removing noise while preserving brain signals (Jin et al., 2023). Motion artifacts from subject movements introduce abrupt signal changes and frequency distortions, which can be mitigated using motion sensors, marker-based corrections, or adaptive filtering.

2. **Handling high-dimensional features in EEG data analysis**: EEG's high temporal resolution and multiple channels create a high-dimensional feature space, leading to the curse of dimensionality. This increases computational complexity and overfitting risks, reducing generalization performance. Selecting relevant features is challenging due to noise, redundancy, and inter-channel dependencies. Traditional feature selection methods are not always effective, and specialized techniques such as filter-based or wrapper-based solutions are needed (Xi et al., 2024). In addition, EEG data captures temporal brain activity, requiring extraction of meaningful temporal features. Time-frequency analysis or wavelet transforms can help preserve temporal dynamics and improve analysis.

3. **In-depth analysis of inter- and intra-subject variability in EEG datasets**: EEG datasets often have limited subject diversity and recording sessions, complicating the ability to capture inter- and intra-subject variability. Variations in EEG signals across individuals and sessions–due to differences in signal quality, electrode contact, and brain activity–can lead to overfitting and poor generalization (Ramezani-Kebrya et al., 2023; Li et al., 2024). Covariate shift, where the distribution of input features changes between subjects or sessions, also poses a challenge. Addressing this variability and covariate shift is essential for developing robust, adaptable models for EEG classification across different subjects and environments.

## 7 Conclusion

This paper introduces a novel framework for EEG classification that integrates PSD and VG features with four deep learning architectures into a unified, end-to-end solution. Through extensive experiments across diverse datasets, we demonstrate the effectiveness of this approach in enhancing classification accuracy. Our model achieved significant improvements over baseline methods. For instance, on the Schizophrenia's dataset, our model achieved an accuracy of 96%, compared to 80% with baseline models, indicating a clear improvement. These quantitative improvements demonstrate that the integration of PSD and VG features, along with the model architecture, leads to more accurate and reliable EEG classification. Looking forward, our framework lays the groundwork for future research in developing more robust EEG-based systems. The integration of PSD and VG features with deep learning models holds promise for a variety of applications, such as autism spectrum disorder analysis, brain tumor diagnostics, and other neurodevelopmental or neurological conditions. This approach contributes to advancing the understanding of complex EEG signals, with potential benefits in both mental health and cognitive neuroscience.

## Data Availability

The original contributions presented in the study are included in the article/supplementary material, further inquiries can be directed to the corresponding author.
